# Nursing staff fatigue and burnout during the COVID-19 pandemic in Greece

**DOI:** 10.3934/publichealth.2022008

**Published:** 2021-11-23

**Authors:** Christos Sikaras, Ioannis Ilias, Athanasios Tselebis, Argyro Pachi, Sofia Zyga, Maria Tsironi, Andrea Paola Rojas Gil, Aspasia Panagiotou

**Affiliations:** 1 Nursing Department, Sotiria Thoracic Diseases Hospital of Athens, 11527 Athens, Greece; 2 Department of Endocrinology, “Elena Venizelou” Hospital, 11521 Athens, Greece; 3 Psychiatric Department, Sotiria Thoracic Diseases Hospital of Athens, 11527 Athens, Greece; 4 Department of Nursing, University of Peloponnese, 22100 Tripoli, Greece

**Keywords:** fatigue, burnout, nurses, COVID-19

## Abstract

**Introduction:**

The coronavirus pandemic (COVID-19) is an unprecedented global health crisis with emotional and physical impact on health care workers.

**Objective:**

The purpose of this study was to investigate the levels of fatigue and burnout in nursing staff during the pandemic.

**Methods:**

The present study involved nursing staff from hospitals in Greece in February 2021, who completed the Fatigue (FAS) and Burnout (CBI) questionnaires. Gender, age, years of work experience, workplace (COVID-19 or non-COVID-19 wards) and SARS-CoV-2 infection status were recorded.

**Results:**

The sample included 593 women and 108 men, with a mean age ± SD: 42.9 ± 9.9 years and 18.14 ± 10.8 years work experience. Slightly more than half, (367, 52.4%) worked in COVID-19 departments. Fifty-six (8%) tested positive for SARS-CoV-2 and 14 of them needed to be treated. The mean ± SD FAS and CBI scores were 25.6 ± 7.4 and 46.9 ± 18.8, respectively (67.9% and 42.9% had scores suggestive of fatigue and burnout, respectively). Women showed higher values in both scales (p < 0.01). Subjects working in COVID-19 wards scored significantly higher on both the FAS and CBI scales; they were also younger and with less work experience (p < 0.01). Staff treated for COVID-19 scored higher on the burnout scale (p < 0.01) than the uninfected staff. Fatigue showed a strong positive correlation with burnout (p < 0.01, r = 0.70). Stepwise multiple regression showed that the variation of fatigue was explained by 47.0% and 6.1% by the scores on the subscales of personal and work-related burnout, respectively.

**Conclusion:**

In conclusion, high rates of fatigue and burnout were found in the studied population. Nurses working with COVID-19 patients had higher rates of fatigue and burnout compared to those working elsewhere. There was a strong positive correlation (r = 0.70) between burnout and fatigue. Particular attention should be paid to staff who became ill and need to be treated.

## 1. Introduction

The novel coronavirus-19 (COVID-19) pandemic is an unprecedented global health crisis [1], which has led to increased depression, anxiety, or worsening of existing mental health issues; its emotional and physical impact is pronounced in healthcare workers (HCW) [2].

For HCW, the strains of professional and social life, as well as the occupational risks associated with exposure to the virus, lead them to increased physical and mental fatigue as well as burnout [2].

Fatigue is medically described as a condition, which is characterized by reduced ability to work as well as reduced performance that follows a period of mental or physical activity [3]. Fatigue that is related to the work of nursing staff has been recognized as a threat to their health but is also negatively associated with patients' safety and quality of care received [4],[5]. It is a complex and multidimensional state with emotional, physiological, cognitive, mental and sensory components that arise as a result of excessive work demands and insufficient energy recovery [5].

In a recent meta-analysis, during the COVID-19 pandemic, the cumulative prevalence of burnout in HCW was 37.4% [6]. Burnout refers to an occupational syndrome associated with emotional and cognitive changes, including emotional exhaustion, depersonalization or cynicism, and decreased feelings of personal effectiveness resulting from chronic work stress [7]. According to Schaufeli & Greenglass, burnout is defined as “a state of physical, emotional and mental exhaustion resulting from long-term involvement in work situations that are emotionally demanding” [8].

Both fatigue and burnout can lead to a feeling of mental and physical exhaustion. Fatigue can be caused by various factors from lifestyle to the environment, while burnout is due to prolonged periods of emotional stress and frustration [9]. The World Health Organization identifies them as two distinct conditions in ICD-10. It recognizes fatigue as a disease and includes it in the codes F48.0 (Neurasthenia) or R53 (Malaise and fatigue) while occupational exhaustion is included in the code Z 73.0 (Burn-out) [9]. It is a prevailing issue in the nursing sector especially during the uncertainty of a pandemic that requires social distancing, more time for using personal protective equipment and changes in the way health services are provided. For practicing clinical nurses, the unavailability of breaks during shifts increases the incidence of fatigue and exhaustion and may result in mental exhaustion. [10],[11].

Similarly, in nursing staff in Greece, the possibility of fatigue and burnout was particularly likely due to lack of personal protective equipment, staff shortages, increased workload and significant risk of infection with the virus. It is worth noting that at the time of the study, the Greek population was in the fourth month of the second lockdown. At that time approximately 1500 new cases of coronavirus were recorded daily, 300–350 patients were hospitalized in intensive care units and 25–30 deaths per day were attributed to COVID-19, with an increasing trend, which was difficult to handle by the national health system. There are studies that show the extent of fatigue and burnout of health care staff during the pandemic, however there is a gap in the literature on infected staff; this study is trying to fill this. Other studies have shown that chronic fatigue is responsible for burnout. We tried to reverse the question and examine whether the previous existence of burnout contributed to current fatigue in nursing staff.

## 2. Objective

The purpose of this study was to evaluate the prevalence of fatigue and burnout in relation to occupational characteristics of nursing staff in Greek public hospitals during the COVID-19 pandemic.

Further research questions were posed:

1. Is there a correlation between fatigue and burnout?

2. Were the nursing staff caring for patients with COVID-19 different in terms of levels of fatigue and burnout compared to the staff caring for patients with other diseases?

3. Were the nursing staff who became ill and hospitalized due to COVID-19 different in terms of levels of fatigue and burnout compared to the staff who were not treated?

## 3. Materials and methods

### 3.1. Study design and setting

This was a descriptive correlational study. Data was collected through anonymous self-report questionnaires, which were forwarded by email. Nurses' email addresses were retrieved through links to websites of Greek nursing/scientific/professional societies. On the first page of the electronic questionnaire, it was clearly indicated that the completion and submission of the questionnaire were considered as a statement of consent. Participation in the research was voluntary. The sample of the study was the nursing staff of Greek public hospitals who responded to the email, convenience sample.

### 3.2. Sample

With a Confidence Level of 99%, a margin of error of 5%, p = 50% and a target population of 27,103 nurses, the minimum study sample was set at 651 subjects.

In total, 701 nurses working in public hospitals throughout Greece agreed and completed the anonymous questionnaires of the 980 to which it was sent (response rate 71.53%). Participants worked in COVID-19 and non-COVID-19 wards, surgical departments, intensive care units (ICUs), or other locations during February 2021. In the invitation for the participation of nurses in the study, an effort was made to make the sample representative of all Greek nurses in terms of gender, geographical distribution of participants, and level of education. Demographic data from study participants included gender and age. Occupational information included department, years of work experience, occupational disease with SARS-CoV-2 (PCR test+), and hospitalization due to COVID-19 in the last six months.

### 3.3. Measurement tools

#### 3.3.1. Fatigue assessment scale (FAS)

The FAS consists of 10 questions (e.g. “I am bothered by fatigue”); each is scored from 1 to 5. Answers include “never, sometimes, often, quite often, always”. Five questions reflect physical fatigue and 5 questions (questions 3 and 6–9) mental fatigue. Every question must be answered, even if the person is not complaining of fatigue. Total scores can range from 10 to 50, with values ≥22 indicating fatigue. The FAS questions aim to capture the fatigue of the last few weeks [12]–[14].

The Greek version of the FAS was used in the present study. The internal consistency, as indicated by Cronbach's alpha, was 0.761 [15].

#### 3.3.2. Copenhagen burnout inventory (CBI)

The CBI is a tool for measuring personal and occupational burnout, consisting of 19 questions and including three subscales:

I. Personal exhaustion [assesses the degree of physical and psychological exhaustion the person experiences; questions 1–6]. It refers to both the physical and psychological exhaustion that accumulates in a person during the day, (e.g. “How often do you feel physically exhausted?”).

II. Work-related exhaustion [assesses the degree of physical and psychological exhaustion the individual perceives about work; questions 7 to 13]. Describes work-related exhaustion (e.g. “Is your job emotionally exhausting?”).

III. Patient-related exhaustion [assesses the degree of physical and psychological exhaustion that is considered by the individual to be related to interaction with patients; questions 14–19]. It depicts exhaustion as a consequence of the interpersonal relationships with patients (e.g. “Does working with patients absorb your energy?”) [16].

Answers include “always, often, sometimes, rarely, and never/almost never” or “to a very high degree, to a high degree, somewhat, to a low degree and to a very low degree”. Each question is scored separately as a continuous variable. The response options are coded in scores of 100, 75, 50, 25, and 0. The items within each subscale are then averaged. Higher scores indicate a higher degree of exhaustion. The possible rating ranges for all subscales are 0–100 [16]. The Greek version of the CBI was used in the present study. The Greek version of CBI is a valid inventory with good psychometric properties. In reliability analysis, Cronbach's alpha exceeds 0.7 for all subscales indicating a high level of internal consistency [17]. In one study, researchers selected scores of 50 or higher to indicate burnout as a dichotomous variable [18], while in another study, researchers selected scores of 25 or lower, 25–50, and higher than 50 to categorize low, intermediate, and high burnout [19]. In the present study, a score of ≥50 (because we wanted to increase the specificity of the questionnaire) was considered as indicative of burnout.

### 3.4. Procedure and ethical considerations

The Ethical Committee of the University of Peloponnese approved the study protocol (2021/01/18). The developer of the FAS granted permission to use the instrument. The developer of the CBI questionnaire Greek version granted permission to use the instrument.

### 3.5. Statistical analysis

All variables were evaluated using descriptive statistics and values were expressed as the mean ± SD for continuous variables. The prevalence of fatigue and burnout was determined as a percentage. Independent *t*-tests were done to evaluate continuous variables by gender. Analyses of variance (ANOVA) with Bonferroni's correction were used to test differences between groups in continuous variables. Pearson's correlation was used to determine correlations between continuous variables and the Partial correlation test. Gradual linear regression analysis was performed to evaluate the continuous variables. Assessment of the linear regression assumptions (linear relationship, independence, homoscedasticity and normality) was done by visual inspection of the variables xy plots, residuals' plots and Q-Q plots. Statistical significance was set at p < 0.05 (two-tailed) and the analyses were performed using IBM SPSS Statistics 23 (IBM SPSS Statistics for Windows, Version 23.0. Armonk, NY: IBM Corp.). Mediation analysis was done with JASP 0.15 (Amsterdam, University of Amsterdam, JASP Team, 2021).

## 4. Results

About 50% of the nursing staff who took part in the study worked in Athens and the rest in regional hospitals. The geographical distribution of the nursing staff of the study was approximately the same as that of the total nursing workforce of the country [20]. In terms of gender, years of work, and age, there was no statistically significant difference compared to the total nursing workforce of the country. In this study Cronbach alpha was for FAS = 0.849 and for CBI = 0.933. However, there was a significant difference in the level of education; in this study, more nurses participated than nursing assistants via-à-vis the composition of the total nursing workforce in the country.

In total, 701 nurses (593 women and 108 men) completed the study questionnaires. A total of 52.4% (367) of the nurses stated they work in a ward with patients with SARS-CoV-2 disease (Table 1).

**Table 1.** General characteristics of nursing staff.

Sex		Age	Work experience (in years)
Nursing staff caring for patients with COVID-19		
Male N = 50	Mean	43.28	17.98
	SD	10.83	11.17
Female N = 317	Mean	41.14	16.66
	SD	9.74	10.60
Total N = 367	Mean	41.43	16.84
	SD	9.91	10.67
Nursing staff caring for patients with non-COVID-19		
Male N = 58	Mean	45.55	20.01
	SD	8.21	9.24
Female N = 276	Mean	44.16	19.48
	SD	10.13	11.15
Total N = 334	Mean	44.40	19.57
	SD	9.83	10.83

The demographic characteristics and work experience of the study participants are presented in Table 2, 67.9% showed a positive score in FAS (≥22) and 42.9% in CBI (personal burnout 54%, work-related burnout 56.8%, patient-related burnout 33.1% ≥50).

**Table 2.** General characteristics of nursing staff and FAS/CBI scores with regards to gender.

Participants	Descriptive statistics	Age	Work experience (in years)	Fatigue assessment scale	Copenhagen burnout inventory			
					Total	Personal burnout	Work-related burnout	Patient-related burnout
Men N = 108	Mean	44.50	19.07	22.58**	39.19**	39.43**	43.18**	34.30*
	SD	9.53	10.18	6.94	18.78	19.55	22.02	22.13
Women N = 593	Mean	42.54	17.97	26.15**	48.36**	51.43**	53.68**	39.07*
	SD	10.03	10.93	7.31	18.42	19.21	21.97	22.93
Total N = 701	Mean	42.85	18.14	25.61	46.95	49.58	52.06	38.33
	SD	9.97	10.83	7.37	18.75	19.73	22.28	22.86

Note: *p < 0.05 or **p < 0.01.

The No statistically significant difference was observed between men and women in terms of age and years of work experience (p > 0.05). Women showed statistically higher averages in both the FAS (independent *t*-test, p < 0.01, 26.15 VS 22.58 mean) and CBI (independent *t*-test, p < 0.01, 48.36 VS 39.19 mean) versus men (Table 2).

These nurses were younger (independent *t*-test, p < 0.01, 41.4 VS 44.4 mean) with fewer years of work (independent *t*-test, p < 0.01, 16.82 VS 19.57 mean) but averaged higher on both the FAS (independent *t*-test, p < 0.01, 26.64 VS 24.47 mean) and the CBI (independent *t*-test, p < 0.01, 49.46 VS 44.18 mean) against nurses who continued to work in wards without patients with COVID-19 disease (Table 3). Statistically higher values were shown by nurses in COVID-19 departments in all three subscales of the CBI (p < 0.05).

**Table 3.** General characteristics of nursing staff and FAS/CBI scores with regards to workplace.

Participants	Descriptive statistics	Age	Work experience (in Years)	Fatigue assessment scale	Copenhagen burnout inventory			
					Total	Personal burnout	Work-related burnout	Patient-related burnout
Staff in a COVID-19 department N = 367	Mean	41.43	16.82	26.64**	49.46**	51.80**	55.56**	40.01*
	SD	9.91	10.67	7.45	18.53	19.03	21.29	23.02
Staff in a non-COVID-19 department N = 334	Mean	44.40	19.57	24.47**	44.18**	47.14**	48.22**	36.49*
	SD	9.83	10.83	7.12	18.64	20.21	22.75	22.57

Note: *p < 0.05 or **p < 0.01.

Fifty-six nurses (8%) stated they tested positive for COVID-19, while 14 nurses (2%) needed to be hospitalized (Table 4). The nurses who needed to be hospitalized did not show a statistical difference either in age (F = 1.19) or in years of work (F = 1.88) from the nurses who tested positive or negative (p > 0.05). On the FAS, nurses in need of hospitalization averaged 27.14 ± 7.15 versus 25.58 ± 7.41 for COVID-19 negative participants and 27.55 ± 6.89 for positive nurses who did not need to be hospitalized, a difference that was not statistically significant (p > 0.05, F = 0.09). In contrast, the nurses who were hospitalized showed a higher CBI score compared to the rest (F = 4.43, Bonferroni p < 0.05).

**Table 4.** General characteristics of nursing staff and FAS/CBI scores with regards to COVID-19 status.

Participants	Descriptive statistics	Age	Work experience (in years)	Fatigue assessment scale	Copenhagen burnout inventory
COVID-19 (–) staff N = 645	Mean	42.73	17.98	25.58	46.46
	SD	10.07	10.90	7.41	18.61
Hospitalized COVID-19 (+) staff N = 14	Mean	48.00	23.93	27.14	61.56
	SD	5.32	6.08	7.16	14.99
Staff positive for COVID-19 N = 42	Mean	42.93	18.71	25.55	47.55
	SD	9.34	10.52	6.86	20.53

To test whether there is a linear relationship between the variables we used scatter plots. The FAS score showed a strong positive correlation with the CBI score (p < 0.01, r = 0.704). Age and years of work showed a weak positive correlation with the CBI sub-scale Patient-related burnout (Table 5).

**Table 5.** Correlations among age, work experience (in years), FAS, CBI.

N = 701 Variables	Age	Work experience (in years)	Fatigue assessment scale	Copenhagen personal burnout	Copenhagen work-related burnout
Work experience (in years)	0.92**				
Fatigue assessment scale	−0.06	−0.03			
Copenhagen burnout inventory (Total)	0.03	0.072	0.70**		
Copenhagen personal burnout	−0.04	−0.01	0.69**		
Copenhagen work-related burnout	−0.01	0.03	0.67**	0.74**	
Copenhagen patient-related burnout	0.12**	0.17**	0.47**	0.50**	0.62**

Note: *p < 0.05 or **p < 0.01.

To further assess factors that influence the FAS's score, we used stepwise multiple regression. The assumptions for linear regression were met.

We defined FAS as the dependent variable and as independent variables: the work experience, the personal CBI burnout, work-related CBI burnout, the patient-related CBI burnout, and whether or not the nurse worked in a COVID department. We checked this model for the absence of multilinearity (variance inflation factor: 2.195 for Copenhagen personal burnout and 2.195 for Copenhagen work-related burnout).

Stepwise multiple regression analysis was performed to identify the best predictors of FAS. This regression showed that 47.0% of the variation of FAS score is explained by the CBI-personal burnout subscale, with an additional 6.1% explained by CBI-work-related burnout. The other variables were not involved in explaining the variance of the FAS (Table 6).

**Table 6.** Stepwise multiple regression (only statistically significant variables are included).

Dependent variable: Fatigue assessment scale	R square	R square change	Beta	t	p
Copenhagen personal burnout	0.470	0.470	0.417	10.85	0.01*
Copenhagen work-related burnout	0.531	0.061	0.364	9.486	0.01*

Note: Beta = Standardized Regression Coefficient; Correlations are statistically significant at the *p < 0.01 level.

With partial correlations we checked the relationships between age, years of work and CBI-patient-related burnout. Controlling for age, the correlation between years of work and CBI-patient-related burnout remained particularly high (p < 0.01). Controlling for working years, the relationship between age and CBI-patient-related burnout was appreciably reduced (p > 0.01).

**Figure 1.**
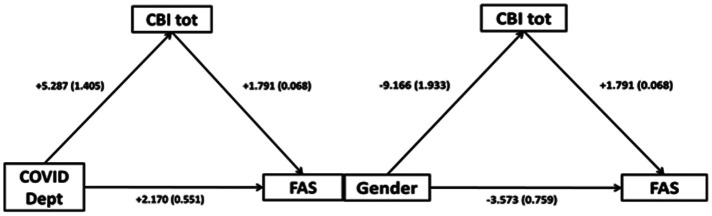
Mediation analysis diagram for workplace (COVID-19/non-COVID-19), gender (men vs women), CBI tot and FAS; regression coefficients with standard errors in parentheses.

The effect of COVID-19/non-COVID-19 workplace on FAS was mediated via CBI tot. More in detail, the indirect effect of COVID-19/non-COVID-19 workplace on FAS, mediated via CBI tot, was b = 1.447 (95% confidence interval [CI]: 0.686 to 2.207, p < 0.001, Sobel test statistic: 3.725, Standard Error [SE]: 2.542, p < 0.0001). Furthermore, the effect of gender (men vis-a-vis women) on FAS was mediated via CBI tot. The indirect effect of gender, mediated via CBI tot, was b = −2.449 (CI: −3.549 to −1.450, p < 0.001, Sobel test statistic: −4.669, SE: 3.515, p < 0.0001) (Figure 1).

## 5. Discussion

The cataclysmic changes the pandemic has brought to our health systems are forcing us to rethink the concept of fatigue and its relationship to burnout. The results of the present study showed a high prevalence of fatigue and burnout among nurses working in public hospitals in February 2021 during the pandemic. The prevalence of fatigue was higher in nurses caring for patients with COVID-19 than in those caring for patients with other diseases. The special conditions of hospital work for patients with an unknown disease (with social and psychological repercussions) can justify this finding. Nursing staff were reported as having relatively high levels of fatigue [21] even before COVID-19. Working at night, in rolling or extended shifts, with reduced amounts and quality of sleep [22]–[24], resulted in fatigue, excessive sleepiness during the day, and reduced work efficiency [25]–[27]. Our results lend credence to studies from China, Spain, Italy, and the United States [4],[22],[28]–[30], which show high rates of fatigue and burnout [22],[31] in nursing staff during the COVID-19 pandemic, as well as in systematic studies prior to COVID-19, which had shown that nursing staff worldwide had moderate to high levels of burnout [32]–[34]. In COVID area younger and less experienced women scored higher in FAS and CBI than the rest of the study participants this finding can be explained by the fact that younger nurses were the ones sent in the first place to Covid areas. Levels of burnout and fatigue were higher among women compared to men, with the most significant gap in personal and burnout.

These findings are consistent with reviews and meta-analyses, which showed that younger female nurses with less clinical experience are more vulnerable to adverse mental health effects [34],[35].

Although burnout has been extensively researched vis-à-vis depression [36] or even alexithymia [37], little is known about its association with fatigue. To the best of our knowledge, there are no new relevant studies examining this relationship, during the pandemic. Our study indicated a strong positive correlation between these two parameters. In our regression analysis, more than half of the FAS fluctuation was explained by the scores of the two CBI scales (personal and work-related burnout). Mediation analysis showed strong mediation of burnout by fatigue. More in detail, it was shown that fatigue and burnout were intertwined and reciprocally mediated any effects of COVID-19/non-COVID-19 workplace or gender on them.

It is worth noting that our study included a significant percentage (8%) of nurses who became infected with SARS-CoV-2 and/or who needed to be treated for COVID-19. Little is known about the psychological burden that infection leaves on health professionals. This is more pertinent, regarding nurses in our study who had to be treated, often in the hospital departments where they worked. They showed higher levels of fatigue (although not statistically significant) but mainly higher burnout. This finding may be explained by the fact that fatigue is a common symptom of post-COVID-19 [38] and by the mediation of burnout by fatigue. It is urgently necessary to create interventions and studies aimed at this particularly vulnerable group.

The aftershock of the pandemic will be felt for a long time. The pronounced fatigue and burnout in nursing staff as a consequence of the pandemic must be addressed immediately. To reduce the psychological impact of the COVID-19 pandemic on nurses, a number of studies and meta-analyses highlight the positive role of interventions such as planning programs to enhance self-efficacy, prevention of PTSD symptoms [39],[40], interventions for improving resilience [41], and implementation of positive behavior strategies [42].

### 5.1. Limitations of this study

There were some important limitations to our study such as that due to the COVID-19 pandemic, it was not possible to distribute the questionnaires in printed form to ensure greater representativeness, although participants responded from all hospitals in the country (from the country's capital and the periphery).

Furthermore, more nurses than nurses' assistants participated in the study, in contrast to the composition of the nursing workforce in the country, although in terms of gender, years of work, and age there was no statistically significant difference.

## 6. Conclusions

Nurses working with COVID-19 patients have high rates of fatigue and burnout. There is an immediate need to address the problem both with administrative measures such as an increase in staff and psychological interventions. A strong positive correlation between burnout and fatigue was noted. Particular attention should be paid to staff who become ill and need to be treated. Because the effects of a pandemic can be long-term, adequate support must continue beyond the pandemic period.
